# Supplementary material for: Spontaneous shock waves in pulse-stimulated flocks of Quincke rollers
Source: Nat Commun. 2023 Nov 3;14:7050. doi: 10.1038/s41467-023-42633-4 (PMC10624688; doi:10.1038/s41467-023-42633-4)
Supplement: Supplementary file 1 — Supplementary Information [file 41467_2023_42633_MOESM1_ESM.pdf]

Supplementary Materials for

Spontaneous shock waves in pulse-stimulated flocks of Quincke rollers

Bo Zhang[1], Andreas Glatz[1,2], Igor S. Aranson[3,4,5], and Alexey Snezhko[1]

[1]Materials Science Division, Argonne National Laboratory, Lemont, IL 60439, USA
[2]Department of Physics, Northern Illinois University, DeKalb, IL 60115, USA
[3]Department of Biomedical Engineering, Pennsylvania State University, University Park, PA 16802, USA
[4]Department of Chemistry, Pennsylvania State University, University Park, PA 16802, USA
[5]Department of Mathematics, Pennsylvania State University, University Park, PA 16802, USA

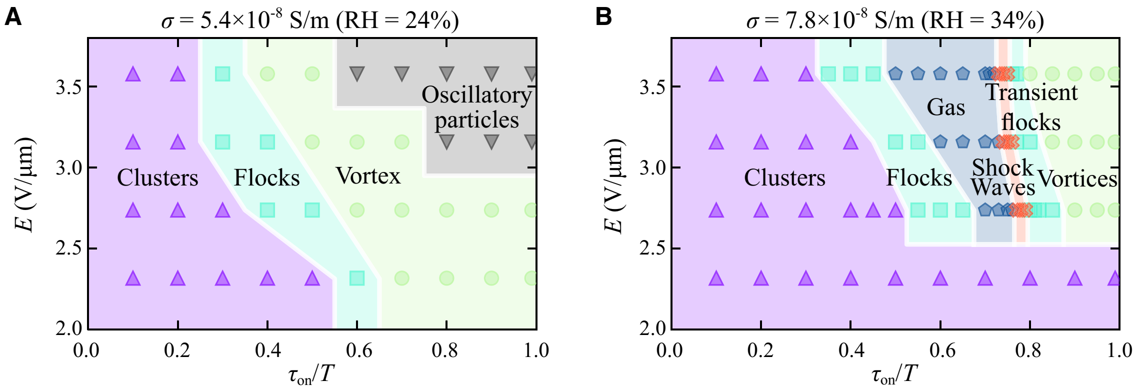

Supplementary Fig. 1: Phase diagrams of rollers at different $\tau_{\rm on}$ and E for low (A) and high (B) conductivities of the media. $T = 6.7$ ms. $\phi_0 = 0.11$. The salt concentration in AOT/hexadecane solution is kept at 0.15 mol/L, while the trace amount of water dissolved is tuned by changing the relative humidity (RH) of the environment. The environment temperature is kept at 23 °C. Any data are collected after the system reaches the dynamically equilibrium state and the conductivity σ does not change.

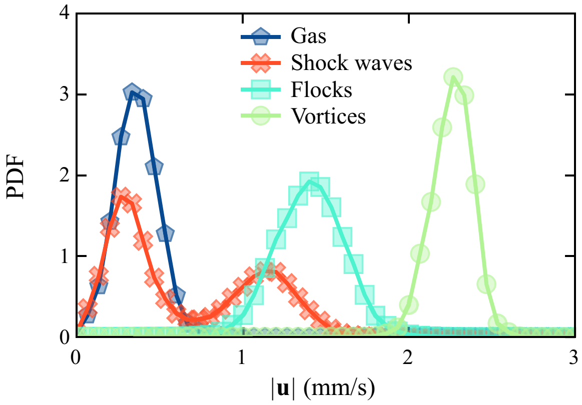

Supplementary Fig. 2: Typical probability distribution functions (PDFs) of the particle speed in different phases: ripples ($\tau_{\rm on} = 4.0$ ms; $\tau_{\rm off} = 2.7$ ms), shock waves ($\tau_{\rm on} = 4.9$ ms; $\tau_{\rm off} = 1.8$ ms), flocks ($\tau_{\rm on} = 5.3$ ms; $\tau_{\rm off} = 1.4$ ms) and vortices ($\tau_{\rm on} = 6.6$ ms; $\tau_{\rm off} = 0.1$ ms). The period is kept at $T = 6.7$ ms.

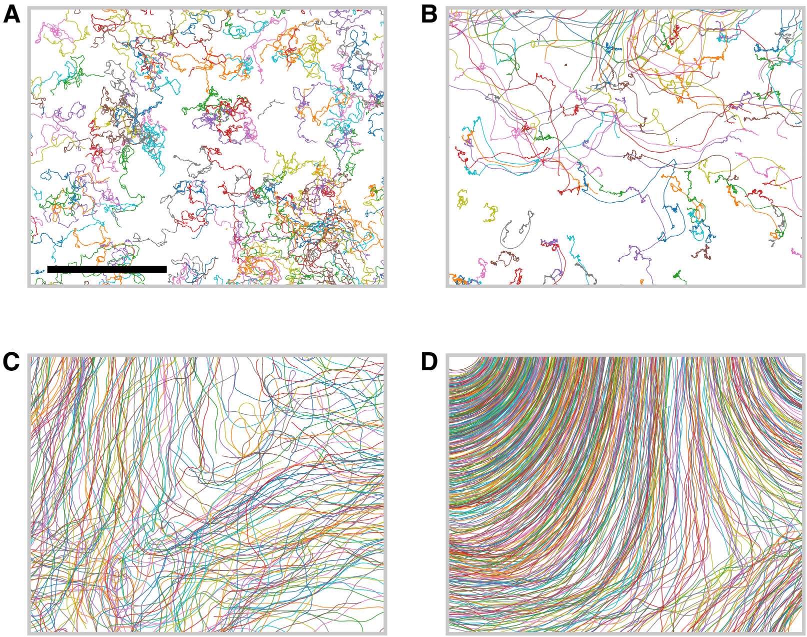

Supplementary Fig. 3: Typical particle trajectories in different phases: ripples (A, $\tau_{\rm on}$ = 4.0 ms; $\tau_{\rm off}$ = 2.7 ms), shock waves (B, $\tau_{\rm on}$ = 4.9 ms; $\tau_{\rm off}$ = 1.8 ms), flocks (C, $\tau_{\rm on}$ = 5.3 ms; $\tau_{\rm off}$ = 1.4 ms) and vortices (D, $\tau_{\rm on}$ = 6.6 ms; $\tau_{\rm off}$ = 0.1 ms). The period is kept at T = 6.7 ms. Only 1 % of particle trajectories are shown.

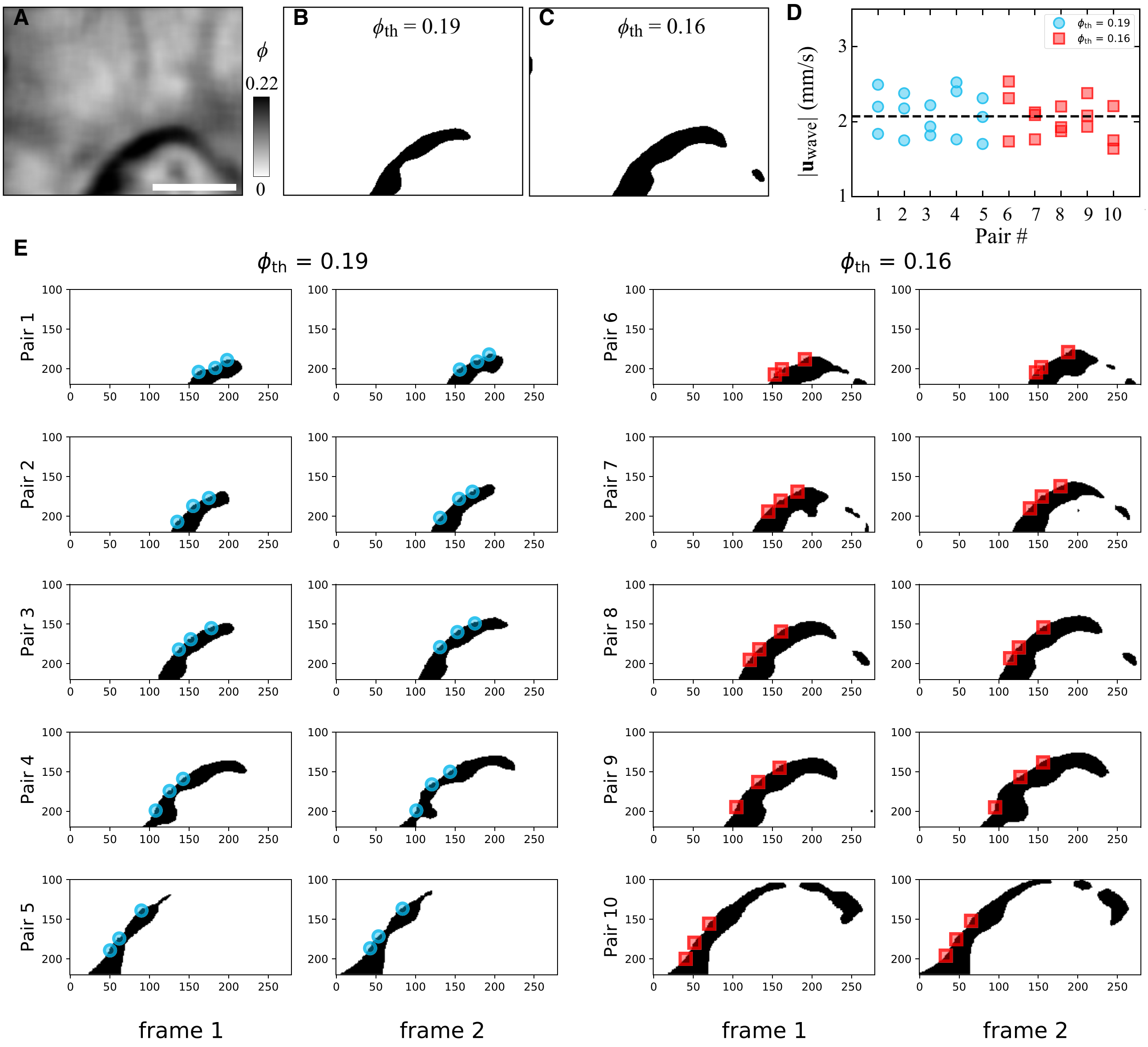

Supplementary Fig. 4: Measurements of the speed of shock waves. (A) Particle density map of a typical shock wave. Since high density regions coexist with high speed regions of show waves as shown in Fig. 2, the shock waves can be presented as high density regions. $E = 3.2$ V/μm. $\phi_0 = 0.11$. $\sigma = 7.8 \times 10^{-8}$ S/m. (B-C) Shock waves identified by two different threshold values of area fractions. (D) Wave speed measured based on the propagation of wave fronts shown in (E). The dash line shows the average speed of 2.1 mm/s.
