# Supplementary material for: Spontaneous shock waves in pulse-stimulated flocks of Quincke rollers
Source: Nat Commun. 2023 Nov 3;14:7050. doi: 10.1038/s41467-023-42633-4 (PMC10624688; doi:10.1038/s41467-023-42633-4)
Supplement: Supplementary file 3 — Description of Additional Supplementary Files [file 41467_2023_42633_MOESM3_ESM.pdf]

Supplementary movie 1

File Name: *Supplementary_Movie_1.mp4*
Description: Ripples. The electric field strength $E = 3.2$ V/µm. $\tau_{\text{on}} = 4.0$ ms; $\tau_{\text{off}} = 2.7$ ms; $T = 6.7$ ms. The frame size is 1.5 mm by 1.2 mm.

Supplementary movie 2

File Name: *Supplementary_Movie_2.mp4*
Description: Multiple shock waves. The electric field strength $E = 3.4$ V/µm. $\tau_{\text{on}} = 4.2$ ms; $\tau_{\text{off}} = 1.4$ ms; $T = 5.6$ ms. The frame size is 2 mm by 1.5 mm.

Supplementary movie 3

File Name: *Supplementary_Movie_3.mp4*
Description: A single shock wave. The electric field strength $E = 3.2$ V/µm. $\tau_{\text{on}} = 4.9$ ms; $\tau_{\text{off}} = 1.8$ ms; $T = 6.7$ ms. The frame size is 1.5 mm by 1.2 mm.

Supplementary movie 4

File Name: *Supplementary_Movie_4.mp4*
Description:Flocks. The electric field strength $E = 3.2$ V/µm. $\tau_{\text{on}} = 3.4$ ms; $\tau_{\text{off}} = 3.3$ ms; $T = 6.7$ ms. The frame size is 1.5 mm by 1.2 mm.

Supplementary movie 5

File Name: *Supplementary_Movie_5.mp4*
Description: Vortices. The electric field strength $E = 3.2$ V/µm. $\tau_{\text{on}} = 6.6$ ms; $\tau_{\text{off}} = 0.1$ ms; $T = 6.7$ ms. The frame size is 3.2 mm by 2.6 mm.

Supplementary movie 6

File Name: *Supplementary_Movie_6.mp4*
Description: Excitation of a shock wave. The electric field strength $E = 3.2$ V/µm. $\tau_{\text{on}} = 5.1$ ms; $\tau_{\text{off}} = 1.6$ ms; $T = 6.7$ ms. The frame size is 1 mm by 1 mm.

Supplementary movie 7

File Name: *Supplementary_Movie_7.mp4*
Description: 2D movie of shock waves in simulations.

Supplementary movie 8

File Name: *Supplementary_Movie_8.mp4*
Description: 3D movie of shock waves in simulations.

Supplementary movie 9

File Name: *Supplementary_Movie_9.mp4*
Description: Ripple free state at low conductivity. The driving field parameters correspond to those in movie S1. The electric field strength $E = 3.2$ V/µm. $\tau_{\text{on}} = 4.0$ ms; $\tau_{\text{off}} = 2.7$ ms; $T = 6.7$ ms. The frame size is 2 mm by 2 mm.

Supplementary movie 10

File Name: *Supplementary_Movie_10.mp4*
Description: Ripple-free gas state. The electric field strength $E = 3.2$ V/µm. $\tau_{\text{on}} = 20$ ms; $\tau_{\text{off}} = 40$ ms.
